# Does Adding an IPACK Block to the Suprainguinal Fascia Iliaca Block Improve the Quality of Analgesia in Patients Undergoing Knee Arthroplasty under Spinal Anesthesia? A Retrospective Cohort Study

**DOI:** 10.3390/medicina59101870

**Published:** 2023-10-20

**Authors:** Hatice Selcuk Kusderci, Caner Genc, Şenay Canikli Adiguzel, Nizamettin Güzel, Serkan Tulgar, Mustafa Suren, Ersin Koksal

**Affiliations:** 1 Department of Anesthesiology and Reanimation, Faculty of Medicine, Samsun Education and Research Hospital, Samsun University, Barış Bulvarı No. 199, 55090 Samsun, Turkey; drkusderci2@gmail.com (H.S.K.); drcanergenc@gmail.com (C.G.); senaycanikli@gmail.com (Ş.C.A.); mustafa.suren@samsun.edu.tr (M.S.); 2 Department of Orthopedics and Traumatology, Samsun Education and Research Hospital, Barış Bulvarı No. 199, 55090 Samsun, Turkey; dr.nizamettinguzel@hotmail.com; 3 Department of Anesthesiology and Reanimation, Ondokuzmayıs University Faculty of Medicine, 55280 Samsun, Turkey; ersin.koksal@omu.edu.tr

**Keywords:** analgesia, arthroplasty, knee, nerve block, pain, regional

## Abstract

*Background and Objectives:* Total knee arthroplasty (TKA) is a commonly performed orthopedic procedure, and is often accompanied by significant postoperative pain. The supra-inguinal fascia iliaca block (SIFIB), similar to an anterior lumbar plexus block, is frequently used in hip surgeries. The interspace between the popliteal artery and capsule of the posterior knee (IPACK) block is a regional anesthesia technique that targets the posterior innervation of the knee capsule. This retrospective study aimed to compare the analgesic effects of SIFIB and SIFIB + IPACK on patients undergoing TKA under spinal anesthesia. *Materials and Methods:* This retrospective study revealed the data collected from a tertiary hospital. Patient data were gathered for individuals who underwent unilateral TKA under spinal anesthesia during the period between 1 January 2023 and 1 September 2023. Inclusion criteria comprised patients falling within ASA class I–III, those following a standardized perioperative analgesia regimen, and individuals receiving opioids via a patient-controlled analgesia device (PCA) as part of their postoperative pain management strategy. Patients were grouped as SIFIB and SIFIB + IPACK according to the performed regional anesthesia technique. *Results:* In the study, the data of 88 patients in total, 61 in the SIFIB group and 27 in the IPACK group, were analyzed. The 24 h cumulative morphine consumption was similar in the SIFIB and SIFIB + IPACK groups (10.62 ± 6.58 mg vs. 12.55 ± 8.84 mg, respectively; *p*: 0.258). The NRS scores of the groups were similar in all time frames. *Conclusions:* Our study reveals that combining IPACK with SIFIB in the multimodal analgesia plan does not provide additional benefits in terms of postoperative opioid consumption and pain scores in patients undergoing unilateral THA under spinal anesthesia.

## 1. Introduction

Total knee arthroplasty (TKA) is a surgical procedure commonly performed to restore the functionality of the knee joint. It is employed worldwide with the primary goal of enhancing patient mobility, alleviating joint discomfort, and improving the overall quality of life for individuals dealing with advanced gonarthrosis [1]. To manage this pain effectively, a comprehensive approach known as multimodal analgesia is used [2]. This approach combines various pharmacological agents such as nonsteroidal anti-inflammatory drugs, opioids, and, on occasion, gabapentinoids, alongside neuraxial techniques. Additionally, interventions such as periarticular injections and peripheral nerve blocks are also incorporated into pain management strategies [3,4].

The knee’s nerve supply is intricately derived from branches originating from both the sacral plexus and lumbar plexus, as highlighted in previous studies [4,5]. Nerves responsible for innervating the surgical field include the femoral nerve (FN), obturator nerve (ON), sciatic nerve (SN), and the lateral femoral cutaneous nerve (LFCN), which is a non-incisional source of pain resulting from tissue manipulation and tourniquet use. These nerves represent potential targets for perioperative pain management strategies [6]. In addition to these nerves, there is ongoing research concerning infiltration techniques specifically targeting the posterior knee capsule, known as the infiltration of the posterior capsule of the knee (IPACK), which has been elucidated and continues to be a subject of investigation [5,6].

Numerous studies and meta-analyses have provided substantial evidence regarding the efficacy of the conventional infra-inguinal fascia iliaca block in alleviating pain associated with knee arthroplasty [2,4]. On the other hand, in studies investigating analgesia in THA, researchers are turning to techniques such as adductor canal blocks, which are relatively distal blocks, due to the lower incidence of quadricep weakness [4,5]. While the application of the supra-inguinal fascia iliaca block (SIFIB) has primarily been confined to hip surgical procedures in existing research, its potential for emerging as a viable analgesic intervention for knee surgeries is noteworthy, given the considerations of its innervation scope and mechanism of action [7]. The rationale behind exploring SIFIB lies in the theoretical understanding that a significant portion of knee innervation originates from the lumbar plexus, potentially leading to results akin to those of lumbar plexus blocks or their components.

However, there is a notable gap in the research concerning the specific application of IPACK as a complementary technique in knee arthroplasty. Our hypothesis posits that incorporating IPACK into the analgesic regimen has the potential to significantly enhance pain management, particularly when considering the neural innervation of the knee’s posterior capsule.

The principal aim of this investigation is to conduct a comparative analysis of opioid consumption within the initial 24 h following surgery in patients who have undergone TKA under spinal anesthesia. Our focus will encompass the evaluation and comparison of patients who have received SIFIB alone against those who have received the combined administration of SIFIB and IPACK. Additionally, secondary objectives encompass the assessment of pain scores and diligent monitoring for the presence of postoperative complications.

## 2. Materials and Methods

For this study, we obtained ethical approval from the Local Ethics Committee (SUKAEK 2023/16/16) and retrospectively analyzed data from patients aged 18 to 75, falling within ASA physiological classes I–III, who underwent unilateral knee arthroplasty under spinal anesthesia at Samsun University Samsun Training and Research Hospital between 1 January 2023 and 1 September 2023. Additionally, this study was registered at clinicaltrial.gov (NCT06054945).

In our clinic, all patients who receive peripheral block techniques as part of the postoperative analgesia plan are diligently monitored and data are recorded on our forms for up to 24 h postoperatively. Detailed information regarding block performance characteristics, complications, postoperative pain intensity at specific time intervals, the presence of motor weakness, and, if PCA is employed, opioid consumption, or, if PCA is not used, rescue analgesic requirements, are meticulously documented and collected. This retrospective study is based on the analysis of meticulously collected data.

The research included patients receiving either a suprainguinal fascia iliaca block (SIFIB) alone or in combination with infiltration of the posterior capsule of the knee (IPACK) for postoperative pain relief. These patients were equipped with patient-controlled analgesia (PCA) devices administering morphine as part of postoperative pain management.

Exclusion criteria comprised individuals with allergies to local anesthetics, injection site infections, those under 18, ASA IV and above patients, and individuals with cognitive impairments affecting pain assessment. All patients underwent uniform spinal anesthesia, perioperative, and postoperative analgesia procedures, with the exception of variations in peripheral block or block combination.

Patient demographics including age, gender, weight, height, and surgery duration were gathered from the data system. Data related to postoperative pain management, such as block concentration, time to initial analgesia request, pain intensity at specified intervals, and analgesic usage at designated time points, were routinely collected in forms, and the primary data for the study were obtained from patients’ follow-up forms. This form only includes data from the first 24 h postoperation.

### 2.1. Standardized Anesthesia Management and Analgesia Plan

*This study was conducted based on retrospective data analysis. In these sections, standard anesthesia/analgesia protocols applied in our clinic are described. Data from patients deviating from standard practices have been excluded.*

All patients are subjected to standard monitoring procedures, including noninvasive arterial blood pressure, electrocardiography, and pulse oximetry. Spinal anesthesia is induced using 12–15 mg of heavy bupivacaine, without any adjuvant, administered via a midline approach targeting the L4–L5 interval.

Comprehensive preoperative, perioperative, and postoperative analgesia protocols are uniformly applied to all patients. Upon the completion of surgery, each patient receives intravenous (IV) tenoxicam (20 mg) and IV acetaminophen (1 g). The postoperative analgesic regimen comprises IV tenoxicam administered every 12 h and IV paracetamol administered every 8 h.

In this retrospective study, the data were limited to patients who were exclusively administered intravenous patient-controlled analgesia (PCA) in the recovery room. In our clinical practice, the PCA system had been configured with no basal infusion and included a solution containing morphine at a concentration of 0.3 mg/mL, with a total volume of 100 mL. Patients had the option to self-administer a bolus dose of 1 mg of morphine through the PCA system, and there was a mandatory lockout period of 20 min between successive doses. PCA initiation occurred immediately following the completion of the nerve block procedure, and patients were instructed to activate the system when their numeric rating scale (NRS) pain score reached or exceeded 4.

We routinely documented the total amount of opioid consumed by patients at specific intervals during the postoperative period, namely at the 1st, 3rd, 6th, 12th, 18th, and 24th hours after the surgery. In cases where, within the first 24 h, the patient reported an NRS pain score of ≥4 despite using PCA, we administered a 25 mg intravenous meperidine injection as rescue analgesia.

***The pain assessment in our clinic was conducted as follows:*** Pain levels experienced by patients were assessed under two conditions: at rest (referred to as NRS-S for static pain) and during motion (referred to as NRS-D for dynamic pain). We recorded NRS-S scores at the 1st, 3rd, 6th, 9th, 12th, 18th, and 24th hours postoperation, while NRS-D scores were documented at the 9th, 12th, 18th, and 24th hours postoperation. The NRS is a unidimensional measure of pain intensity used in adults. It involves patients rating pain on a numerical scale comprising eleven points, ranging progressively from no pain (0) to the most severe pain imaginable (10).

***Evaluation of Quadriceps weakness:*** To assess the weakness of the quadricep muscle, the patient’s knee is extended actively. This is carried out initially against the force of gravity and subsequently against applied resistance, all while keeping the hip joint flexed at a 45-degree angle and the knee joint flexed at 90 degrees. A 3-point rating scale is used to assess and score the patient’s ability to extend the knee as follows: 0 = normal strength (the patient can extend the knee against both gravity and applied resistance); 1 = mild weakness (the patient can extend the knee against gravity but struggles against resistance); 2 = severe weakness (the patient cannot extend the knee). In statistical evaluations, quadricep weakness is accepted as present (2,1) or absent (0).

### 2.2. Block Performances

In this section, information is given about the application procedures of the regional anesthesia techniques applied in our clinic and the subject of this study.

- ***Ultrasound guided IPACK block***

The ultrasound-guided IPACK block procedure is carried out within a designated regional anesthesia application section situated in a separate area within the operating room, preoperatively. Prior to the block procedure, all patients undergo standard ASA monitoring, and those considered hemodynamically suitable are administered 0.03 mg/kg of midazolam to induce sedoanalgesia. After placing the patient in a prone position, the sequential steps for the IPACK block can be succinctly delineated as follows. The initiation process entailed the scanning of the popliteal fossa using a lower-frequency ultrasound transducer (3–5 MHz, Esaote MyLabTM30Gold, Genoa, Italy). Once the femoral condyle became discernible within the US field, the probe was directed proximally until the visualization of the femoral shaft was achieved, consequently exposing the popliteal artery. Subsequently, a nerve block needle (Vygon Echoplex, 85 mm, 21 G, Ecouen, France) was introduced, originating from the lateral aspect of the knee joint, targeting the region positioned between the popliteal artery and the femur, followed by the administration of 20 mL of 0.25% bupivacaine.

- ***Ultrasound guided SIFIB***

In our clinic, we apply SIFIB at the end of surgery in order to benefit from its analgesic effectiveness for a longer period of time. The US-guided SIFIB is administered postoperatively after the successful completion of a surgical procedure, once patients are transferred to the postanesthetic care unit, undergo standard ASA monitoring, and are observed to be hemodynamically stable. With the patient positioned supine, the inguinal region is aseptically prepared, and a sterile linear US transducer (10–18 MHz, Esaote MyLabTM30Gold, Genoa, Italy) is situated adjacent to the inguinal ligament, aligning its longitudinal axis parallelly to the ligament. The femoral artery and the femoral nerve are distinctly identified. Subsequently, (2) the probe is laterally repositioned to locate the sartorius muscle, ensuring that the image of the sartorius muscle is centrally displayed on the US screen. The probe is then moved cephalad, tracking the sartorius muscle until it gradually disappears while the probe traverses towards the hypoechoic anterior superior iliac spine. The US probe undergoes a 90° rotation to identify the anterior superior iliac spine, the iliacus muscle, and the abdominal muscles, all of which are visualized on the screen. The needle is introduced from the caudal aspect and directed cranially using an in-plane technique, ultimately allowing the needle tip to perforate the fascia iliaca and reach the fascia iliaca compartment. Following the confirmation of the accurate placement of the needle tip, achieved by injecting 5 mL of saline, a total of 40 mL of 0.25% bupivacaine is administered.

### 2.3. Outcome Measurements

The primary outcome of our retrospective study was the total amount of opioids required within the 24 h period following the procedure. Our secondary outcome measures encompassed the assessment of pain scores at varying intervals. Additionally, we documented ancillary factors such as episodes of nausea, vomiting, the time elapsed until the first request for analgesia, and instances of quadriceps weakness lasting more than 24 h.

### 2.4. Statistics

SPSS software (SPSS, version: 16.0, Chicago, IL, USA) was used for statistical analysis. To assess the distribution of data, we utilized the Kolmogorov–Smirnov test to evaluate normality. Descriptive data were presented using either the mean and standard deviation or the median and interquartile range (25th and 75th percentiles). Continuous variables were compared using either Student’s *t*-test or the Mann–Whitney U test, depending on the data distribution. Proportions were compared using the chi-square test with Yates’ correction, while Fisher’s exact test was utilized for categorical variables such as ASA classification and gender. To determine statistical significance, we set the threshold at *p* < 0.05. Notably, for the analysis of numeric rating scale (NRS) scores measured at five different time points, we applied the Bonferroni correction, with statistical significance defined as *p* < 0.001.

## 3. Results

Between the specified dates, data from 61 SIFIB and 27 SIFIB + IPACK patients who met the inclusion criteria for unilateral knee surgery were analyzed. The mean ages of the patients were 65.82 ± 4.97 years in the SIFIB group and 64.29 ± 3.95 years in the SIFIB + IPACK group, and these data were similar (*p*: 0.163). Additionally, in terms of descriptive data such as gender distribution, ASA class, length, weight, BMI, and other parameters, the groups were similar (*p* > 0.05), (Table 1). The patients’ first analgesic request times were also similar in the SIFIB and SIFIB + IPACK groups (5.09 ± 2.90 vs. 4.92 ± 2.44 h, respectively, *p* > 0.05).

**Figure 1:**
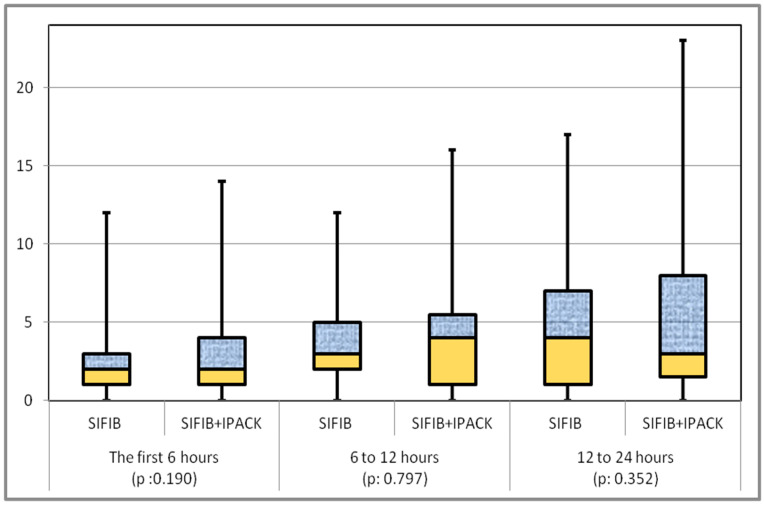
Box plot demonstration and comparison of opioid consumption in different time periods.

When comparing the cumulative morphine consumption of the groups at the 24th hour, it was 10.62 ± 6.58 mg in the SIFIB group and 12.55 ± 8.84 mg in the SIFIB + IPACK group, with no statistically significant difference observed (*p*: 0.258) (Table 2). Furthermore, cumulative morphine consumption between the groups was similar in other time frames. Additionally, there were no significant differences in morphine requirements between the groups during the first 6 h, 6–12 h, and 12–24 h periods (Figure 1).

**Figure 2:**
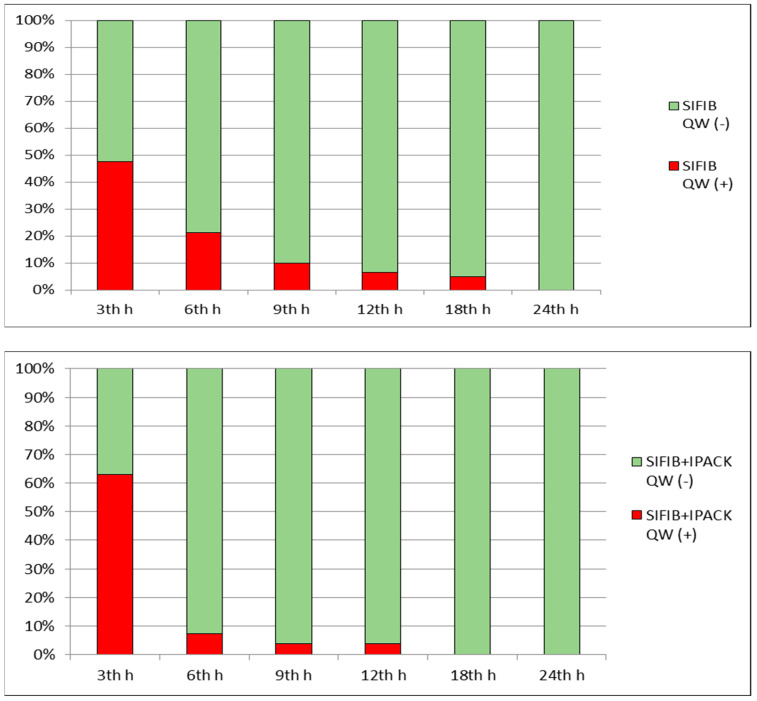
Percentage distribution of patients with and without quadriceps weakness according to groups (QW: Quadriceps weakness).

When comparing the groups in terms of pain scores, NRS scores at rest and with movement were similar in all time frames (*p* < 0.05), (Table 3). In both groups, no motor weakness lasting more than 24 h was identified. The percentage rates of patients with and without quadricep weakness are presented in detail in Figure 2. Additionally, there were no patients requiring meperidine as a rescue analgesia other than PCA and scheduled analgesia.

## 4. Discussion

In this retrospective study, we found that there was no statistical difference in opioid requirement and pain scores between the SIFIB alone and the combination of the SIFIB and IPACK as the regional component of multimodal analgesia in patients undergoing unilateral TKA under spinal anesthesia. Furthermore, there was no difference observed in terms of the time to the first analgesic request and the rate of quadricep weakness. Thus, we were unable to establish any superiority of one technique over the other in any aspect.

When considering skin incision for TKA, it is observed that the entirety of sensory innervation originates from the lumbar plexus [3,8]. Similarly, when taking into account knee capsule and osseous cuts, the majority of innervation, except for the posterior aspect of the knee capsule, derives from the lumbar plexus [9]. The posterior capsule of the knee is innervated by the popliteal plexus, which in turn receives branches from the deep branch of the obturator nerve and the sciatic nerve [5,10,11].

As a complementary approach to ensuring perioperative analgesia in patients undergoing knee surgery, the IPACK block can be effectively combined with other techniques. In the current literature on knee surgery, more distal blocks such as the ACB are preferred for analgesia purposes in knee surgeries—at the expense of narrowing the sensory block area—due to there being less motor blocking. When combined with an adductor canal block (ACB), it can provide high-quality perioperative analgesia in patients undergoing TKA [12]. However, Chatmaitri et al. [13] demonstrated that the addition of an IPACK block to patients undergoing TKA with ACB + PAI (periarticular infiltration) did not confer an additional benefit to pain management. On the other hand, Laoruengthana et al. [14] compared the application of IPACK + ACB in patients undergoing total knee arthroplasty with the surgically administered PAI. They reported that both groups provided similar analgesia results in the initial 48 h; however, after 72 h, the ACB + IPACK group reported a higher intensity of pain. In their meta-analysis, Guo et al. [12] demonstrated that the addition of an adductor canal block to IPACK resulted in decreased postoperative VAS scores, reduced cumulative morphine consumption, and shortened hospital stays. However, Tang et al. [15] conducted a meta-analysis indicating that adding the IPACK block to patients undergoing total knee arthroplasty (TKA) who had already received multimodal analgesia for perioperative pain management exhibited an effect on pain scores, morphine consumption, and functional recovery supported by moderate-level evidence. Therefore, in this study, our aim was to determine whether or not adding IPACK to the SIFIB in patients undergoing TKA would alter the quality of postoperative pain.

The obturator nerve contributes to the posterior innervation of the knee by joining the popliteal plexus with its deep branches (articular branches) along with the sciatic nerve. Therefore, the application of SIFIB may contribute to analgesia not only in the anterior but also in the posterior aspect of the knee, facilitated by obturator nerve blockade. Given the existing research on the efficacy of ACB and IPACK combinations, there is a discernible gap to be filled via more extensive investigations into novel techniques and technical alternatives. An exploration of motor block incidence at different SIFIB volumes and concentrations represents a promising avenue for future studies. Additionally, potential research may involve a comparative analysis of quadricep weakness and analgesic efficacy between distal blocks such as ACB and ACB + IPACK, and proximal blocks such as SIFIB in TKA.

Vermeylen et al. [16] demonstrated that in the SIFIB technique, the local anesthetic exhibited a more consistent spread beneath the fascia iliaca and around the psoas muscle in a cranial direction, allowing for the better targeting of the three main components of the lumbar plexus through an anterior approach. Amshi et al. [17] reported the development of quadriceps muscle weakness in four patients who underwent the SIFIB. It should be noted that when applying SIFIB in patients undergoing same-day surgery and requiring early mobilization, the potential for motor blockade should be considered. Furthermore, the distance from anatomically significant vascular structures and the ease of application in the supine position are among the other significant advantages of the SIFIB.

In TKA, instead of blocking the lumbar plexus from a high level, the general trend is to block it from a more distal level (such as adductor canal block and genicular block) and avoid motor weakness [3]. In our study, we found that motor weaknesses were observed very rarely for more than 12 h, and perhaps this rate could be further reduced with lower concentrations. Of course, there is a need for studies comparing distal blocks and block combinations with high blocks such as SIFIB.

There are some limitations of our study. Regarding the constraints of our study, it encompasses elements related to a retrospective approach, including data insufficiency, the challenge of establishing causal links, the potential for confounding factors from data sources to be present, limitations in group selection and the oversight of temporal influences, inherent design restrictions, data variability, and potential biases. These limitations underscore the need for the prudent interpretation of our results and caution regarding the definitiveness of our study’s findings. Additionally, the most crucial limitation at this point is the lack of studies in the literature demonstrating the effectiveness of the SIFIB on pain management in patients undergoing TKA. However, in a randomized controlled study conducted by our clinic very recently, it was reported that the SIFIB reduced the postoperative analgesic requirement [18]. In this study, it was determined that SIFIB application reduced the morphine requirement by 50% compared to that of the control group.

## 5. Conclusions

Our study showed that adding IPACK to the SIFIB, which is a regional anesthesia technique in the multimodal analgesia plan, has no additional benefit in terms of postoperative opioid consumption, pain scores, the first analgesic request, and the rate of quadriceps weakness in patients undergoing unilateral THA under spinal anesthesia.

## Figures and Tables

**Table 1:** Comparison of descriptives and time until the first need for analgesia between groups.

	SIFIB (*n*: 61)	SIFIB + IPACK (*n*: 27)	*p*
Age (years)	65.82 ± 4.97	64.29 ± 3.95	0.163
Gender F/M	51/10	22/5	0.806
ASA II/III	55/6	25/2	0.649
Length	161 ± 5.97	161.5 ± 8.25	0.778
Weight	78.24 ± 2.88	76.22 ± 8.41	0.247
BMI	30.23 ± 2.88	29.30 ± 3.38	0.189
First analgesic demand (h)	5.09 ± 2.90	4.92 ± 2.44	0.791

Data are expressed as mean ± standard deviation or number. The first analgesic demand time was determined from the end of surgery. *p* values are italicized and *p* values that are written in bold represent statistical significance.

**Table 2:** Comparison of cumulative morphine consumption at different time points.

Cumulative Morphine Consumption	SIFIB (*n*: 61)	SIFIB + IPACK (*n*: 27)	*p*
3rd h	0 (0–1)	0 (0–1)	0.348
6th h	2 (1–3)	2 (1–4)	0.190
9th h	3 (2–7)	4 (2–9)	0.590
12th h	5 (3–8)	6 (2–12)	0.375
18th h	6 (4–11)	8(3–13.5)	0.252
24th h	10 (5–15)	10 (5–19.5)	0.258

The data are expressed as medians (percentiles 25–75). *p* values in bold indicate statistical significance.

**Table 3:** Comparison of numeric rating scores at different time points between SIFIB and SIFIB + IPACK.

NRS-at Rest (Static)	SIFIB (*n*: 61)	SIFIB + IPACK (*n*: 27)	*p*
1st h	0 (0–0)	0(0–1)	0.448
3rd h	1 (0–2)	2 (1–3)	0.660
6th h	2 (1–3)	2 (2–2)	0.746
9th h	2 (1–2)	2 (1–2)	0.668
12th h	2 (1–2)	2 (1–2.5)	0.716
18th h	1 (1–2)	2 (1–2)	0.690
24th h	1 (1–2)	2 (1–2)	0.102
**NRS-at Movement (Dinamic)**	**SIFIB** **(*n*: 61)**	**SIFIB + IPACK** **(*n*: 27)**	***p***
1st h	0 (0–1)	0 (0–1)	0.888
3rd h	2(1–3)	2 (2–3)	0.497
6th h	3 (2–4)	3 (3–3)	0.784
9th h	3 (2–3)	3 (2–3)	0.400
12th h	3 (2–3)	3 (2–3)	0.308
18th h	2 (2–3)	3 (2–3)	0.131
24th h	2 (2–3)	2 (2–3)	0.224

Data are expressed as median (percentiles 25–75). NRS: numeric rating score.

## Data Availability

Data are available upon request from the corresponding author.

## References

[1] Heo K., Karzon A., Shah J., Ayeni A., Rodoni B., Erens G.A., Guild G.N., Premkumar A. (2023). Trends in Costs and Professional Reimbursements for Revision Total Hip and Knee Arthroplasty. J. Arthroplast..

[2] Joshi G.P., Stewart J., Kehlet H. (2022). Critical appraisal of randomised trials assessing regional analgesic interventions for knee arthroplasty: Implications for postoperative pain guidelines development. Br. J. Anaesth..

[3] Tran D.Q., Salinas F.V., Benzon H.T., Neal J.M. (2019). Lower extremity regional anesthesia: Essentials of our current understanding. Reg. Anesth. Pain Med..

[4] Fillingham Y.A., Hannon C.P., Kopp S.L., Austin M.S., Sershon R.A., Stronach B.M., Meneghini R.M., Abdel M.P., Griesemer M.E., Woznica A. (2022). The Efficacy and Safety of Regional Nerve Blocks in Total Knee Arthroplasty: Systematic Review and Direct Meta-Analysis. J. Arthroplast..

[5] Sankineani S.R., Reddy A.R.C., Eachempati K.K., Jangale A., Gurava Reddy A.V. (2018). Comparison of adductor canal block and IPACK block (interspace between the popliteal artery and the capsule of the posterior knee) with adductor canal block alone after total knee arthroplasty: A prospective control trial on pain and knee function in immediate postoperative period. Eur. J. Orthop. Surg. Traumatol..

[6] Shroff J.B., Hanna P., Edgar C.M. (2023). Surgeon-Directed Arthroscopic Infiltration Between the Popliteal Artery and Capsule of the Knee (IPACK) Block: Technical Description. Arthrosc. Tech..

[7] Sanllorente-Sebastián R., Arroyo-García B., Avello-Taboada R. (2020). Ultrasound suprainguinal fascia iliaca block in knee surgery. J. Anesth..

[8] Laurant D.B.S., Peng P., Arango L.G., Niazi A.U., Chan V.W.S., Agur A., Perlas A. (2016). The nerves of the adductor canal and the innervation of the knee: An anatomic study. Reg. Anesth. Pain Med..

[9] Tran J., Peng P.W.H., Lam K., Baig E., Agur A.M.R., Gofeld M. (2018). Anatomical Study of the Innervation of Anterior Knee Joint Capsule: Implication for Image-Guided Intervention. Reg. Anesth. Pain Med..

[10] Runge C., Moriggl B., Børglum J., Bendtsen T.F. (2017). The Spread of Ultrasound-Guided Injectate from the Adductor Canal to the Genicular Branch of the Posterior Obturator Nerve and the Popliteal Plexus: A Cadaveric Study. Reg. Anesth. Pain Med..

[11] Tran J., Peng P.W.H., Gofeld M., Chan V., Agur A.M.R. (2019). Anatomical study of the innervation of posterior knee joint capsule: Implication for image-guided intervention. Reg. Anesth. Pain Med..

[12] Guo J., Hou M., Shi G., Bai N., Huo M. (2022). iPACK block (local anesthetic infiltration of the interspace between the popliteal artery and the posterior knee capsule) added to the adductor canal blocks versus the adductor canal blocks in the pain management after total knee arthroplasty: A systematic review and meta-analysis. J. Orthop. Surg. Res..

[13] Chatmaitri S., Tangwiwat S., Halilamien P., Ruangsomboon P., Pornrattanamaneewong C., Chareancholvanich K., Narkbunnam R. (2022). Efficacy of adding an interspace block to the posterior knee for perioperative pain in total knee arthroplasty: A randomized controlled trial. Acta Orthop..

[14] Laoruengthana A., Rattanaprichavej P., Kositanurit I., Saenghirunvattana C., Samapath P., Pongpirul K. (2022). Adductor Canal Block Combined with Interspace between the Popliteal Artery and Capsule of the Knee (iPACK) versus Periarticular Injection for Total Knee Arthroplasty. Clin. Orthop. Surg..

[15] Tang X., Lai Y., Du S., Ning N. (2022). Analgesic efficacy of adding the IPACK block to multimodal analgesia protocol for primary total knee arthroplasty: A meta-analysis of randomized controlled trials. J. Orthop. Surg. Res..

[16] Vermeylen K., Desmet M., Leunen I., Soetens F., Neyrinck A., Carens D., Caerts B., Seynaeve P., Hadzic A., Van de Velde M. (2019). Supra-inguinal injection for fascia iliaca compartment block results in more consistent spread towards the lumbar plexus than an infra-inguinal injection: A volunteer study. Reg. Anesth. Pain. Med..

[17] Vamshi C., Sinha C., Kumar A., Kumar A., Kumari P., Kumar A., Kumar S., Arun S.K. (2023). Comparison of the efficacy of pericapsular nerve group block (PENG) block versus suprainguinal fascia iliaca block (SFIB) in total hip arthroplasty: A randomized control trial. Indian J. Anaesth..

[18] Kefeli Çelik H., Tulgar S., Güler S., Koç K., Küçükordulu B.B., Ferli R.B., Kehribar L., Genç A.S., Süren M. (2023). Evaluation of Postoperative Analgesic Efficacy of Ultrasound-Guided Suprainguinal Fascia Iliaca Block in Knee Arthroplasty: Prospective, Randomized, Feasibility Study. J. Clin. Med..

